# Deferoxamine Treatment Improves Antioxidant Cosmeceutical Formulation Protection against Cutaneous Diesel Engine Exhaust Exposure

**DOI:** 10.3390/antiox10121928

**Published:** 2021-11-30

**Authors:** Erika Pambianchi, Francesca Ferrara, Alessandra Pecorelli, Mascia Benedusi, Hina Choudhary, Jean-Philippe Therrien, Giuseppe Valacchi

**Affiliations:** 1 Department of Animal Science, Plants for Human Health Institute, North Carolina State University, Kannapolis, NC 28081, USA; epambia@ncsu.edu (E.P.); apecore@ncsu.edu (A.P.); 2 Department of Environment and Prevention, University of Ferrara, 44121 Ferrara, Italy; frrfnc@unife.it (F.F.); mascia.benedusi@unife.it (M.B.); 3 SkinCeuticals, New York, NY 10001, USA; hina024@gmail.com; 4 JP Therrien Consulting, LLC, 17107 Arkansas Ln, Davidson, NC 28036, USA; jptherrien@hotmail.com; 5 Department of Food and Nutrition, Kyung Hee University, Seoul 02447, Korea

**Keywords:** cosmeceuticals, pollution, iron-chelator

## Abstract

Skin is one of the main targets of the outdoor stressors. Considering that pollution levels are rising progressively, it is not surprising that several cutaneous conditions have been associated with its exposure. Among the pollutants, diesel engine exhaust (DEE) represents one of the most toxic, as it is composed of a mixture of many different noxious chemicals generated during the compression cycle, for ignition rather than an electrical spark as in gasoline engines. The toxic chemicals of most concern in DEE, besides the oxides of nitrogen, sulfur dioxide and various hydrocarbons, are metals that can induce oxidative stress and inflammation. The present study aimed to evaluate the effects of topical application, singularly or in combination, of the iron-chelator deferoxamine and a commercially available formulation, CE Ferulic, in up to 4-day DEE-exposed skin. DEE induced a significant increase in the oxidative marker 4-hydroxy-nonenal (4HNE) and matrix-metallopeptidase-9 (MMP-9), the loss of cutaneous-barrier-associated proteins (filaggrin and involucrin) and a decrease in collagen-1, while the formulations prevented the cutaneous damage in an additive manner. In conclusion, this study suggests that iron plays a key role in DEE-induced skin damage and its chelation could be an adjuvant strategy to reinforce antioxidant topical formulations.

## 1. Introduction

The World Health Organization (WHO) estimated that circa 90% of the urban population around the world lives in areas with pollutant levels that exceed WHO guideline limits, and this has been linked to the premature death of 7 million people yearly [1].

The use of the word “pollution” can be misleading given that are several the different pollutants to which living organisms are exposed. Based on their chemical and physical properties, the USDA has divided them into six main groups: ozone (O_3_), particulate matter (PM), carbon monoxide (CO), lead, sulfur dioxide (SO_2_) and nitrogen dioxide (NO_2_) [2].

Among them, the role of PM on human health has been investigated for several years, and many pathologies have been associated with its exposure. Indeed, it has been shown that PM can impact the structure and functionality of many organs, including lungs, brain, hearth, lungs and gut [3,4,5,6]. Only more recently the association between PM exposure and skin conditions has been analyzed, and now there is solid evidence suggesting the role of PM in accelerating skin aging, increasing cutaneous spots and inducing skin inflammation [7,8].

Among the PM, diesel engine exhaust (DEE) particles have been shown to be among the most noxious to human health and among the most predominant worldwide [9]. Of all existing internal combustion engines, the diesel engine is among the most popular and is therefore of great concern with respect to the environment and public health. Invented in the late 1800s, the diesel engine became very popular in 1980s, and since then, its popularity has continuously expanded and occupied the urban centers, increasing the spread of these toxic compounds in the environment.

Although most of the attention of airborne PM has focused on the impact on human respiratory and cardiovascular systems [10,11] and other organs, such as gut, liver and kidney [12,13], recently the effect of DEE on skin has been investigated. In fact, as a consequence of its peculiar location, cutaneous tissue acts as a biological shield against air pollutants, and prolonged and repetitive exposure to high levels of airborne PM has been shown to have profound adverse effects on the integumentary apparatus [8,14].

Diesel exhaust is a complex mixture composed of a solid core, where, based on its source, several elements, including metals, are present. The harmful effects of DEE exposure have been suggested to involve local Reactive Oxygen Species (ROS) production, which could, in part, be generated from the particles themselves. On the other hand, particles can serve as organic compounds’ carriers, such as polycyclic aromatic hydrocarbons (PAHs), which are highly lipophilic and capable of localizing in mitochondria, contributing to the evidenced ROS production [15].

The oxidative capacity of PM has been attributed to its transition metal constituents, especially iron (Fe), which can catalyze Fenton-like reactions and generate ROS, initiating oxidative damage mechanisms [16,17] by the formation of hydroxyl radicals. Therefore, the presence of iron can be considered as one of the possible triggers of the noxious effect of DEE exposure.

Consequently, to reduce the excess of pro-oxidant metal, iron-chelators, such as deferoxamine (DFO), have been widely used to prevent iron damage. As of today, only a few studies have investigated the role of iron as part of the possible harmful effect of DEE exposure on cutaneous tissues and the possible novel use of the iron-chelators as adjuvant for already established anti-pollution formulations [18,19] to prevent photoaging [19].

Our study aims to evaluate the use of the iron chelator DFO, as a topical treatment, to be combined to antioxidant topical interventions. More specifically, the present work focuses on the evaluation of the possible additive effect of the topical combination of DFO with the commercially available antioxidant cosmeceutical formulation, CE Ferulic, against DEE-induced skin damage.

## 2. Materials and Methods

### 2.1. Human Specimens

Human Caucasian skin explants were purchased from Hunstad/Kortesis/Bharti Cosmetic Surgery clinic and derived from three different healthy subjects (age 35–45) undergoing routine elective abdominoplasties. For each tissue sample, collected following informed consent, skin biopsies were taken with a 12 mm full thickness punch, and subcutaneous fat was trimmed by using sterile scissors. The skin biopsies were then rinsed with Phosphate Buffer Solution (PBS) containing antibiotics/antimycotic and then transferred into 6-well plates that were prefilled with 1 mL of DMEM High Glucose supplemented with 10% Fetal Bovine Serum (FBS), 100 IU/mL penicillin and 100 μg/mL streptomycin (complete medium), using sterile technique [20]. The plates were incubated at 37 °C in a 5% CO_2_/95% air atmosphere and left undisturbed for overnight recovering.

### 2.2. Application of CE Ferulic, Deferoxamine (DFO) and Vehicles

After overnight recovering, skin biopsies medium was replaced with fresh complete medium. Then, 5 μL of CE Ferulic composed by 15% L-ascorbic acid, 1% α-tocopherol and 0.5% ferulic acid (CE Ferulic, SkinCeuticals Inc., Dallas, TX, USA), 20 μL of DFO 100 μM (DM533, Sigma Aldrich), solubilized in PBS, alone or in combination, and corresponding vehicles (CE Ferulic vehicle and PBS) were applied topically to the skin explants, in triplicate for each condition. For the combination CE Ferulic + DFO, first 20 μL of DFO were applied and evenly spread with a sterile glass rod, and then after 15 min (to allow for treatment absorption), 5 μL of CE Ferulic was added and uniformly distributed. The same was performed for the combination of CE Ferulic vehicle and PBS. Plates were incubated in humidified 5% CO_2_/95% air atmosphere for 24 h. The experiment was performed for each donor and at least in triplicates for each condition.

### 2.3. Diesel Engine Exhaust (DEE) Exposure

On the following day, the skin biopsies were treated again with the abovementioned compounds before the exposure to DEE for 30 min. DEE was generated by a Kubota RTV-X900 diesel engine (3-cylinder, 4-cycle diesel with overhead valves, 1123 cc that has 24.8 HP at 3000 rpm), as previously reported [21]. The engine was left to run for 10 s, allowing the diesel exhaust to be delivered to an exposure chamber. The skin explants were then left in the sealed chamber for 30 min, in the presence of DEE. A set of skin explants, controls, were exposed to purified and HEPA-filtered air. After DEE exposure, media were replaced, and tissue samples were incubated in humidified 5% CO_2_/95% air atmosphere and then collected either 24 h after the first exposure (Day 1) or after four days (Day 4). Treatment and exposure were performed daily.

### 2.4. Tissue Collection and Immunohistochemical Analysis

At the end of each time point (Day 1 and Day 4), skin explants were fixed in 10% neutral buffered formalin for 48 h at 4 °C, dehydrated and embedded in paraffin. Then 4 μm–thick sections were deparaffinized in xylene and then rehydrated through a series of decreasing alcohols to water. Antigen retrieval was performed by boiling slices in 10 mM sodium citrate buffer solution (Thermo Fisher Scientific, Waltham, MA, USA) (pH 6.0) at a sub-boiling temperature, in a 500 W microwave, for 10 min. After cooling, slices were washed in PBS; blocked with 5% Bovine Serum Albumin (BSA) in PBS; and incubated with primary antibodies for 4HNE (dil. 1:500) (AB5605, Millipore Corporation, Burlington, MA, USA), collagen 1 (dil. 1:400) (AB34710, Abcam, Cambridge, UK), pro-MMP-9 (dilution. 1:50) (MAB9111, Novus Biologicals, Littleton, CO, USA) MMP-9 (dil. 1:200) (NBP2-13173, Novus Biologicals, Littleton, CO, USA), Involucrin (dil. 1:50) (sc-21748, Santa Cruz Biotechnology, Inc., Dallas, TX, USA) or Filaggrin (dil. 1:50) (sc-66192, Santa Cruz Biotechnology, Inc., Dallas, TX, USA) in 2% BSA in PBS. Sections were then washed three times in PBS and incubated with fluorochrome-conjugated secondary antibodies (dil. 1:500) (Alexa Fluor 568, A11004 or Alexa Fluor 488, A11055) in 2% BSA in PBS at RT, and then washed with PBS. Nuclei were stained with DAPI (D1306, Invitrogen) in PBS. After a triple series of washings with PBS, slices were mounted by using PermaFluor mounting media (ThermoFisher Scientific, Waltham, MA, USA) and imaged on a Zeiss LSM10 microscope, equipped at 40× magnification. Images were quantified by using ImageJ [22].

### 2.5. Statistical Analysis

Statistical analyses were performed via GraphPad Prism 6 software (GraphPad Software, Inc.). Differences between groups were evaluated by analysis of variance (ANOVA), considering time points (Day 1 and Day 4) separately, followed by Tukey’s post hoc test. A *p*-value ≤ 0.05 was considered statistically significant. All variables tested are expressed as mean ± standard deviation (SD) of three independent experiments.

## 3. Results

### 3.1. Evaluation of CE Ferulic and DFO Pretreatment in Preventing DEE Exposure-Induced Cutaneous 4HNE Protein Adducts Formation

Considering that the outermost layer of the skin, the stratum corneum, is rich in lipids, it is a perfect substrate for the interaction with DEE that leads to the formation of several lipid oxidation products (LOPs), among which 4-hydroxy-nonenal (4HNE) is one of the most reactive.

**Figure 1:**
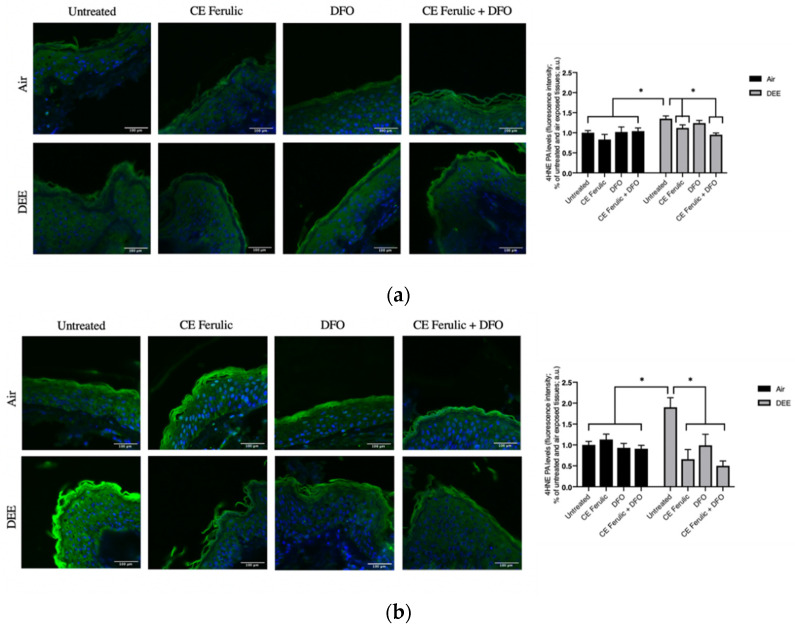
Skin exposure to DEE increases lipid peroxidation related damage, while topical application of CE Ferulic and its combination with DFO can prevent this effect. Levels of 4HNE PA in ex vivo human skin explants untreated or pretreated with the cosmeceutical formulation mixture and DFO, alone or in combination, and exposed to air or DEE at Day 1 (**a**) and Day 4 (**b**). Green fluorescence staining represents 4HNE PA immunoreactivity, while cell nuclei are stained in blue with DAPI. Original magnification 40×. On the right, semi-quantification of the immunofluorescence intensities performed by ImageJ are shown in the histograms. Data are expressed as arbitrary units (averages of three independent experiments), * *p* ≤ 0.05, by ANOVA. Scale bar = 100 μm.

As shown in Figure 1, DEE exposure induces the formation 4HNE protein adducts, with a moderate increase at Day 1 (Figure 1a) and with a more evident and significant induction at Day 4 (Figure 1b). DFO treatment was able to clearly prevent 4HNE formation at Day 4, and CE Ferulic showed the ability to prevent 4HNE formation from Day 1 (Figure 1a) to Day 4 (Figure 1b). The combined application of CE Ferulic and DFO seemed to promote, at both time points, a moderate additive effect in reducing DEE-exposure-induced 4HNE levels (Figure 1a,b).

Collectively, these results confirmed the harmful effect of DEE exposure on cutaneous tissue and propose beneficial additive properties of the combined topical application of the iron chelator DFO and the cosmeceutical formulation CE Ferulic in preventing the formation of lipid peroxidation in exposed skin.

### 3.2. Evaluation of CE Ferulic and DFO Pretreatment in Preventing DEE Exposure Modulation of Pro-MMP-9 and MMP-9 Levels

Different pollutants have been shown to be both the cause and the effect of inflammatory responses, therefore fueling a vicious circle that is referred to as OxInflammation [21,23]. Matrix metallopeptidase 9 (MMP-9) is an enzyme responsible for the degradation and turnover of extracellular matrix components (EMCs) and tissues’ remodeling. It has been shown that MMP-9 is a key player in cutaneous inflammation when its activation is aberrantly overexpressed [24,25,26].

**Figure 2:**
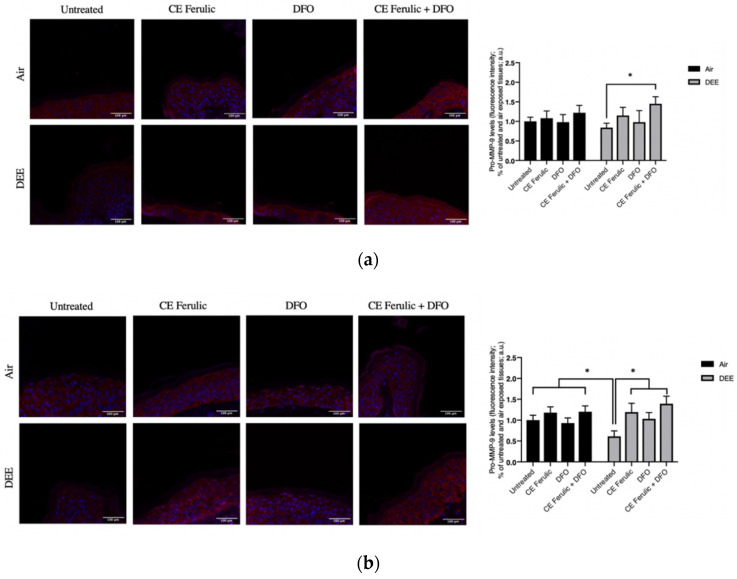
Skin exposure to DEE induces a decrease in the levels of pro-MMP-9, while topical application of CE Ferulic and DFO prevents this effect. Levels of pro-MMP-9 in ex vivo human skin explants untreated or pretreated with the cosmeceutical formulation mixture and DFO, alone or in combination, and exposed to air or DEE at Day 1 (**a**) and Day 4 (**b**). Red fluorescence staining represents pro-MMP-9 immunoreactivity, while cell nuclei are stained in blue with DAPI. Original magnification 40×. On the right, semi-quantification of the immunofluorescence intensities performed by ImageJ are shown in the histograms. Data are expressed as arbitrary units (averages of three independent experiments), * *p* < 0.05, by ANOVA. Scale bar = 100 μm.

**Figure 3:**
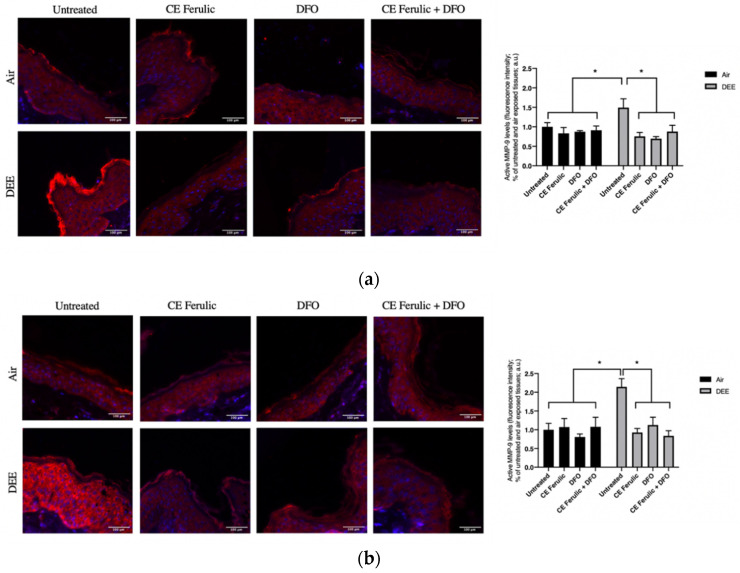
Skin exposure to DEE induces the levels of MMP-9 enzyme, while topical application of CE Ferulic and DFO counteracts this effect. Levels of MMP-9 in ex vivo human skin explants untreated or pretreated with the cosmeceutical formulation mixture and DFO, alone or in combination, and exposed to air or DEE at Day 1 (**a**) and Day 4 (**b**). Red fluorescence staining represents MMP-9 immunoreactivity, while cell nuclei are stained in blue with DAPI. Original magnification 40×. On the right, semi-quantification of the immunofluorescence intensities performed by ImageJ are shown in the histograms. Data are expressed as arbitrary units (averages of three independent experiments), * *p* < 0.05, by ANOVA. Scale bar = 100 μm.

To further evaluate the effect of DEE and topical application of CE Ferulic and DFO, we also measured the MMP9 levels of the pro-form (pro-MMP-9) and the active form (MMP-9). Pro-MMP-9 is an inactive zymogen with an un-cleaved pro-peptide domain that requires further processing by other MMPs or serine proteases to become fully active and yield to EMC degradation. As shown in Figure 2, DEE exposure decreases the levels of pro-MMP-9 at Day 1 (Figure 2a), and this effect becomes even more significant at Day 4 (Figure 2b), suggesting the activation of this enzyme. As a proof of concept, we also measured the levels of the active form of MMP-9. As shown in Figure 3, DEE exposure clearly induced MMP-9 activation in skin at both Day 1 (Figure 3a) and Day 4 (Figure 3b). This effect was completely quenched by the topical application of both DFO and CE Ferulic over time.

The topical application of CE Ferulic and DFO, and, even more, their combination, was able to prevent the cleavage of pro-MMP9 and preventing its activation.

### 3.3. Evaluation of CE Ferulic and DFO Pretreatment in Preventing DEE Exposure Induced Effects on Cutaneous Collagen 1

**Figure 4:**
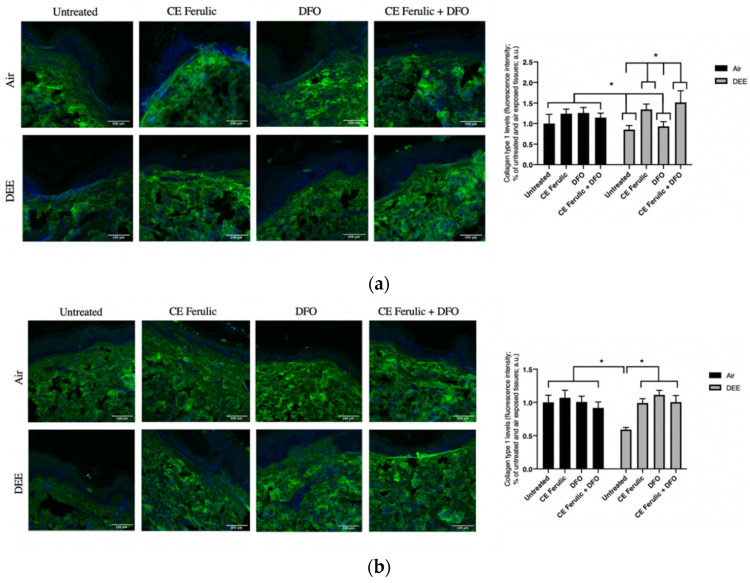
Skin exposure to DEE decreases levels of collagen 1, but topical application of CE Ferulic and DFO prevents this effect. Levels of collagen 1 in ex vivo human skin explants untreated or pretreated with the cosmeceutical formulation mixture and DFO, alone or in combination, and exposed to air or DEE at Day 1 (**a**) and Day 4 (**b**). Green fluorescence staining represents collagen 1 immunoreactivity, while cell nuclei are stained in blue with DAPI. Original magnification 40×. On the right, semi-quantification of the immunofluorescence intensities performed by ImageJ are shown in the histograms. Data are expressed as arbitrary units (averages of three independent experiments), * *p* < 0.05, by ANOVA. Scale bar = 100 μm.

Type I collagen is one of the most abundant molecules in the body, and it is particularly important in skin and in the connective tissue. It is an interstitial matrix collagen organized in fibrils, which are essential for the competence of the skin. Collagen-1 depletion is one of the most evident signs of skin aging, and it has been linked with the cutaneous premature aging induced, among the many factors, also by pollution exposure. As depicted in Figure 4, exposure to DEE significantly decreased the levels of collagen 1 at both time points Day 1 and Day 4. This effect was prevented by CE Ferulic and only slightly by DFO at Day 1 (Figure 4a). After 4 days of exposure, both the compounds were able to significantly prevent the loss of collagen 1, although no additive effect was noticed when DFO and CE Ferulic were applied simultaneously (Figure 4b).

### 3.4. Evaluation of CE Ferulic and DFO Pretreatment in Preventing DEE Exposure Induced Decrease of Cutaneous Filaggrin Levels

**Figure 5:**
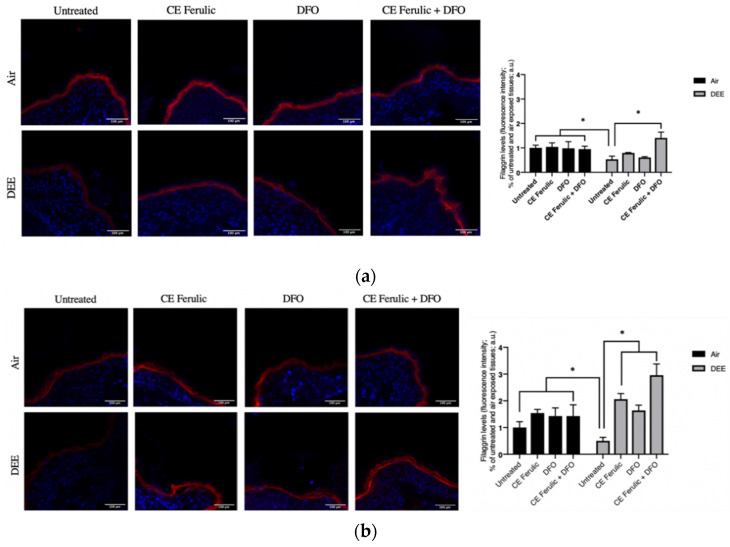
Skin exposure to DEE induces a decrease in filaggrin levels, while topical application of CE Ferulic and DFO prevents this effect. Levels of filaggrin in ex vivo human-skin explants untreated or pretreated with the cosmeceutical formulation mixture and DFO, alone or in combination, and exposed to air or DEE at Day 1 (**a**) and Day 4 (**b**). Red fluorescence staining represents filaggrin immunoreactivity, while cell nuclei are stained in blue with DAPI. Original magnification 40×. On the right, semi-quantification of the immunofluorescence intensities performed by ImageJ are shown in the histograms. Data are expressed as arbitrary units (averages of three independent experiments), * *p* < 0.05, by ANOVA. Scale bar = 100 μm.

The skin barrier is fundamental to ensure healthy skin, and previous studies have demonstrated that exposure to pollutants can exacerbate inflammatory skin diseases by compromising the cutaneous barrier itself and its associated proteins, such as filaggrin [21,27], a protein involved in epidermal hydration and keratin matrix formation [27]. As shown in Figure 5a,b, at both time points (Day 1 and Day 4), there was a significant decrease in filaggrin protein levels after DEE exposure when compared to the untreated tissues exposed to air. The treatments with CE Ferulic and DFO alone prevented filaggrin loss in DEE-exposed tissues at Day 4; moreover, their combined topical application resulted in a clear additive effect in counteracting DEE-induced filaggrin loss. In addition, the combined treatment further induced filaggrin levels over the baseline, suggesting a possible new mechanism to induce this important protein when its levels are seriously compromised [27,28].

### 3.5. Evaluation of CE Ferulic and DFO Pretreatment in Preventing DEE Exposure Induced Decrease of Cutaneous Involucrin Levels

Together with filaggrin, involucrin is another important protein involved in the formation of keratinocytes cornified cell envelope, being therefore a key component of fully differentiated epidermal layer and playing an essential role in skin barrier.

**Figure 6:**
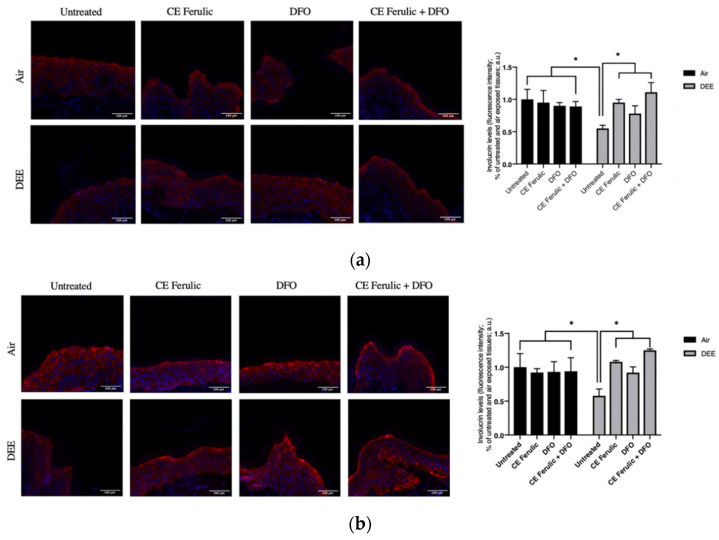
Skin exposure to DEE induces a decrease in involucrin levels, while topical application of CE Ferulic and DFO prevents this effect. Levels of involucrin in human-skin explants untreated or pretreated with the cosmeceutical formulation mixture and DFO, alone or in combination, and exposed to air or DEE at Day 1 (**a**) and Day 4 (**b**). Red fluorescence staining represents involucrin immunoreactivity, while cell nuclei are stained in blue with DAPI. Original magnification 40×. On the right, semi-quantification of the immunofluorescence intensities performed by ImageJ are shown in the histograms. Data are expressed as arbitrary units (averages of three independent experiments), * *p* < 0.05, by ANOVA. Scale bar = 100 μm.

Similarly to the effect of DEE on filaggrin levels, DEE, at both time points (Day 1 and Day 4), remarkably downregulated the levels of involucrin when compared to untreated air-exposed skin explants (Figure 6a,b). This harmful effect was prevented by the topical application of CE Ferulic and DFO. Noteworthy, the combined topical treatment was not only able to prevent DEE decreased involucrin levels, but, as shown also for the filaggrin (Figure 5), it was able to further stimulate its levels over the baseline.

Taken together, these data corroborate the damaging effects induced by DEE on cutaneous tissues and suggest that the combined topical application of CE Ferulic and the iron chelator—in our case, DFO—can strongly improve skin-barrier functions by increasing involucrin and filaggrin levels in DEE-exposed skin.

## 4. Discussion

Diesel engine emissions have increased significantly in the last few decades, particularly in densely populated urban cities and areas. Among the different environmental pollutants, DEE is the most prevalent anthropogenic source of pollution worldwide [9]. Diesel exhaust is a complex mixture, and even if its exact composition is intimately related on the engine type, operation mode, type of fuel, lubrication oil and their respective additives [29,30], three primary fractions can be identified: solid, condensates (hydrocarbons, water and sulfuric acid) and gaseous. The solid fraction is mainly composed by the elemental carbon, metals (such as magnesium, iron, manganese, platinum, copper, zinc and cerium) and non-metal oxides, mainly generated by normal engine wear, lubrication and fuel additives.

In general, exposure to airborne particulate matters (PM) is associated with several skin conditions, including accelerated extrinsic aging [31], pigment-spot formation, coarse-wrinkle development and elastosis [32].

The mechanisms involved in PM-associated skin disorders result from increased oxidative stress due to PM exposure. It is still under debate the ability of PM to penetrate the skin, although it has been suggested that eventually particulate matter is able to move through the skin via hair follicles or trans-dermally, generating oxidative stress [33,34,35].

In addition, polycyclic aromatic hydrocarbons (PAHs), which are components of diesel particles, can be absorbed through the skin and eventually damage the mitochondria, resulting in intracellular ROS production [36]. These damaged mitochondria produce superoxide anions, which can be converted into H_2_O_2_ that can then undergo the Fenton reaction (as a consequence, for instance, of the iron present in the particles), to produce hydroxyl radicals, resulting in increased ROS and activation of redox sensitive transcription factors, such as AP1 and NFκB.

Therefore, the role of metals, and, in particular, of iron as a mediator of diesel particle toxicity, has been postulated. For this reason, the possible usage of iron chelators together with other topical antioxidants mixtures could be a good approach to prevent harmful effects of PM on skin health.

Our data confirmed this hypothesis, as the increase in lipid peroxidation measured by 4HNE levels was induced by DEE exposure and prevented by DFO treatment, and this effect was even more evident when DFO was added together with CE Ferulic. This was in line also with previous findings where the induction of an oxidative stress status in the skin was the consequence of pollutants exposure (alone or in combination) [20,21].

In addition to this oxidative damage, DEE and other environmental pollutants have been demonstrated to exhibit another more specific harmful mechanism via the disruption of iron homeostasis. Air pollutants can induce alterations in the cellular ferrokinetics by either (a) complexing/chelating this micronutrient (via double bonds or the presence of electronegative functional groups) or (b) by displacing it from pivotal cellular sites [37,38].

The first mechanisms can induce the formation of complexes between the pollutant and iron itself, with the ability of reacting as a Fenton’s reagent and catalyzing electron exchange and oxidative stress [39,40]. The same excess iron associated with pollution exposure can complex with available hydrogen and lipid peroxides to generate hydroxy and lipid radicals, resulting in the cellular organelles and structures damage.

At the same time, the cell tries to restore intracellular iron by generating superoxide to allow for the chemical reduction step of ferric iron (Fe^3+^) to ferrous iron (Fe^2+^) required for the intracellular trace mineral entry [41,42,43]. This reduction step triggers a vicious cycle that can generate more ROS, consequently enhancing the release of iron from protein carriers (ferritin) [44]. This second wave of iron restoration could explain the more evident increase in 4HNE after 4 days of exposure, in respect to early time points, as the increase of iron released from ferritin could further augment the cutaneous oxidative status and the formation of LOP.

Increased oxidative stress has been often associated with inflammatory processes, and this crosstalk can lead to a positive vicious-cycle-defined OxInflammation [45], where oxidative stress induced by exogenous factors, e.g., pollution, triggers and, at the same time, is fueled by aberrant inflammatory responses [21,23].

This was confirmed in our study by the increased levels of MMP-9 after DEE exposure. Of note, DFO and CE Ferulic were able to quench MMP-9 activation in an additive manner, suggesting that both iron and ROS are involved in its enzymatic activity.

MMP-9 expression is regulated by redox-sensitive transcription factors (NFkB and AP1), and this enzyme is involved in the turnover and degradation of extracellular matrix components, such as collagen, contributing to wrinkle formation and premature skin aging. Therefore, it was not surprising that DEE exposure was able to affect collagen I levels and that the pretreatment with DFO and CE Ferulic could prevent this damaging effect. These data confirmed the anti-pollution effect of CE Ferulic, as also noticed in a human study aimed to evaluate the cutaneous noxious effect of O_3_ [26].

The oxidation and inflammation induced by DEE exposure can directly harm the skin structure and barrier function, which, on the other hand, can fuel the perpetuation and amplification of the cutaneous OxInflammatory state itself.

In support of this idea, our study demonstrated that filaggrin (an important marker of differentiation and healthy skin-barrier functions) [46] was decreased upon 1 and 4 days of DEE exposure. These data are in line with previous work by Lee et al. [43], who suggested that an inflammatory status can downregulate filaggrin levels. In addition, the same authors have recently shown how the activation of NFkB is involved in keratinocytes filaggrin loss [47]. Our group and others have indeed demonstrated the activation of NFkB by PM in skin models, confirming the hypothesis that the loss of filaggrin could be in part due to the inflammatory status [14].

Our data are also corroborated by a recent work by Li et al. [48], where PM exposure not only decreased filaggrin levels in keratinocytes, but it also significantly affected involucrin levels. The ability of PM to affect important cutaneous markers of differentiation can be interpreted as the ability of pollution to compromise skin barrier properties and possibly further allow external factors (e.g., pathogens, pollutants, etc.) to enter the skin.

Even if several studies demonstrated that skin health can be improved by diet [49], the local topical application should still be considered as a potent ally to prevent pollution-induced cutaneous damage. Supporting this theory is the extensive use of commercially available antioxidant formulation mixtures that have proven to have the ability to protect against single and combined pollutants exposure in preclinical and clinical studies [21,26,50,51].

One of the treatments applied in these studies is represented by the CE Ferulic, which is composed of 15% L-ascorbic acid, 1% α-tocopherol and 0.5% ferulic acid. This mixture, when topically applied, has shown to penetrate the stratum corneum (therefore possibly activating skin defensive mechanisms, such as Nrf2 as demonstrated in 2D and 3D models [45,46]), preventing the UV-induced erythema, p53 activation, sunburn cells and DNA damage [52] and surprisingly exerting an additive beneficial photo-protective effect compared to the one mediated by the single elements [21,53,54,55].

The suggested additive mechanism of action of this formulation is that ferulic acid acts as a reducing agent for vitamin E and C, therefore, as suggested by Pinnel’s work, protecting and elongating their reducing potential [53].

The aim of this study was the evaluation of a possible additive effect of two combined protective elements: a radical scavenger-like CE Ferulic and an iron chelator, such as DFO. At the base of this idea is that, as pollution can induce extreme levels of oxidative stress and consequent lipid peroxidation, the use of antioxidant alone may not be enough to provide protection against DEE-induced skin damage [56]. In fact, the dual action of DEE that is able to induce an unbalanced oxidative status directly and indirectly (via iron) suggests the protection through a bifunctional tool that targets both ROS and iron.

To date, many studies have proved iron chelators, and DFO, in particular, to be valuable allies in preventing iron-mediated oxidative damage. DFO is a hydrophilic drug with a short half-life, and delivery in vivo into the hydrophobic stratum corneum can be somehow difficult; therefore, more work needs to be performed in trying to develop an efficient transdermal delivery system, as suggested by Rodrigues et al. [57]. They were able to uses reverse micelles to deliver DFO and improve wound healing in diabetic models.

Anyway, many other scientific groups demonstrated DFO to be suitable for topical application, specifically to ameliorate cutaneous chronic wounds [58], radiation-induced soft tissue injury [59], alleviate skin injury [60] and to prevent pressure-induced diabetic ulcers [61].

Consistent with these insights are also our data that demonstrated how topically applied DFO, either alone or in combination with CE Ferulic, was able to counteract not only the DEE-induced lipid peroxidation and prevent MMP-9 activation, but also showed protective properties of the skin barrier, specifically by reverting the DEE-induced decrease in the involucrin and filaggrin levels.

However, new scientific insights showed that prolonged treatment with DFO or other effective iron chelators, such as arolhydrazone chelators [62], can provoke severe cellular iron starvation, leading to clinical complications [63,64,65]. Therefore, other innovative iron chelator molecules could be taking the place of the most known DFO.

In particular, novel “smart pro-drugs” iron chelators have demonstrated effective capacities in preventing iron-induced oxidative damage upon UVA exposure and at the same time have shown physicochemical properties that allow them to be selectively activated in situ within the skin, only upon UVA irradiation [66]. These molecules, known as photo-activable and photo-controller “caged” iron chelators could be the next step of topical anti-pollution protection.

## 5. Conclusions

In conclusion, although our research can be considered preliminary, it still allows us to corroborate previous data demonstrating the protective properties of CE Ferulic against pollution-induced cutaneous OxInflammation. Furthermore, it showed that the combined topical application of a cosmeceutical mixture containing CE Ferulic and an iron chelator, such as DFO, could have an additive protective effect against DEE-induced skin damage.

## Data Availability

The data presented in this study are available in this manuscript.
